# Replicating Single-Cycle Adenovirus Vectors Generate Amplified Influenza Vaccine Responses

**DOI:** 10.1128/JVI.00720-16

**Published:** 2017-01-03

**Authors:** Catherine M. Crosby, William E. Matchett, Stephanie S. Anguiano-Zarate, Christopher A. Parks, Eric A. Weaver, Larry R. Pease, Richard J. Webby, Michael A. Barry

**Affiliations:** a Department of Medicine, Division of Infectious Diseases, Mayo Clinic, Rochester, Minnesota, USA; b Virology and Gene Therapy Graduate Program, Mayo Clinic, Rochester, Minnesota, USA; c Clinical Translational Sciences Graduate Program, Mayo Clinic, Rochester, Minnesota, USA; d Immunology Graduate Program, Mayo Clinic, Rochester, Minnesota, USA; e Department of Immunology, Mayo Clinic, Rochester, Minnesota, USA; f Department of Molecular Medicine, Mayo Clinic, Rochester, Minnesota, USA; g Department of Infectious Diseases, St. Jude Children's Research Hospital, Memphis, Tennessee, USA; University of Pittsburgh School of Medicine

**Keywords:** adenovirus, gene-based vaccine, replication defective, single cycle

## Abstract

Head-to-head comparisons of conventional influenza vaccines with adenovirus (Ad) gene-based vaccines demonstrated that these viral vectors can mediate more potent protection against influenza virus infection in animal models. In most cases, Ad vaccines are engineered to be replication-defective (RD-Ad) vectors. In contrast, replication-competent Ad (RC-Ad) vaccines are markedly more potent but risk causing adenovirus diseases in vaccine recipients and health care workers. To harness antigen gene replication but avoid production of infectious virions, we developed “single-cycle” adenovirus (SC-Ad) vectors. Previous work demonstrated that SC-Ads amplify transgene expression 100-fold and produce markedly stronger and more persistent immune responses than RD-Ad vectors in Syrian hamsters and rhesus macaques. To test them as potential vaccines, we engineered RD and SC versions of adenovirus serotype 6 (Ad6) to express the hemagglutinin (HA) gene from influenza A/PR/8/34 virus. We show here that it takes approximately 33 times less SC-Ad6 than RD-Ad6 to produce equal amounts of HA antigen *in vitro*. SC-Ad produced markedly higher HA binding and hemagglutination inhibition (HAI) titers than RD-Ad in Syrian hamsters. SC-Ad-vaccinated cotton rats had markedly lower influenza titers than RD-Ad-vaccinated animals after challenge with influenza A/PR/8/34 virus. These data suggest that SC-Ads may be more potent vaccine platforms than conventional RD-Ad vectors and may have utility as “needle-free” mucosal vaccines.

**IMPORTANCE** Most adenovirus vaccines that are being tested are replication-defective adenoviruses (RD-Ads). This work describes testing newer single-cycle adenovirus (SC-Ad) vectors that replicate transgenes to amplify protein production and immune responses. We show that SC-Ads generate markedly more influenza virus hemagglutinin protein and require substantially less vector to generate the same immune responses as RD-Ad vectors. SC-Ads therefore hold promise to be more potent vectors and vaccines than current RD-Ad vectors.

## INTRODUCTION

Influenza virus is an enveloped, single-stranded, segmented, negative-sense RNA virus from the family Orthomyxoviridae, with three major types (A, B, and C) (1). Influenza A virus is subtyped based on its expressed surface glycoproteins, hemagglutinin (HA) and neuraminidase (NA). There are 16 HA subtypes and 9 NA subtypes. For example, in the 2014-2015 influenza season, most influenza virus infections were due to infections by influenza A virus subtype H3N2 (2, 3). Influenza A viruses infect humans and many other species, including birds, pigs, horses, whales, and seals. Influenza B viruses infect humans and seals. Influenza C viruses infect humans and pigs. This ability to infect multiple species allows influenza viruses to exchange or reassort their RNA segments to produce highly pathogenic viruses that can “jump” between species and cause devastating pandemics.

Influenza virus infections begin at mucosal surfaces. In humans, these infections largely affect the upper and lower respiratory tracts but can also affect other mucosal sites, including the digestive tract (1, 4–8). In contrast, avian influenza viruses manifest primarily as infections in the gut. Regardless of the primary site of disease, all influenza virus infections start at mucosal surfaces in all species, making these critical sites for generating protective immune responses.

Seasonal influenza epidemics in humans are caused by influenza A and B viruses. In a normal year, influenza virus infections may cause over 200,000 hospitalizations and approximately 37,000 deaths in the United States. Worldwide, these infections may account for 3 to 5 million cases of severe illness and 250,000 to 500,000 deaths each year (1, 2, 9). Periodically, the seasonal infections become pandemics that can kill millions.

To combat the pathogen, there have been two types of licensed vaccines. Both vaccines are derived from intact infectious influenza virions. The first is a trivalent (TIV) or quadrivalent (QIV) inactivated vaccine. The second is a trivalent live-attenuated influenza vaccine (LAIV). For both TIV and LAIV, the primary goal of vaccination is to deliver the HA protein to generate antibodies that can neutralize the virus. Ideally, neutralization would occur at mucosal surfaces, where the initial infection starts. In the absence of neutralizing IgA or IgG antibodies at mucosal surfaces, IgG antibodies in body fluids may play a role in controlling the virus after the mucosal barrier is breached. TIV is delivered by the intramuscular route using syringes and needles (10). Because TIV is chemically inactivated, it cannot infect cells, replicate its genome, or amplify antigen production. TIV can therefore generate antibodies and CD4^+^ T cell responses but largely fails to produce antiviral CD8 T cells. TIV also elicits only minimal levels of IgA at mucosal surfaces that are highly correlated with protection from influenza virus infection (4, 5). While TIV is used in 90% of influenza vaccinations, its efficacy is only 59% (9).

LAIV is a “needle-free” vaccine and is administered by direct inoculation into the nose. LAIV was developed from temperature-sensitive influenza virus (11–13). LAIV is able to replicate its genome and its antigens in the cooler noses of vaccine recipients after intranasal administration, but the mutation prevents viral replication in warmer deep tissues, like the lung, thereby avoiding frank influenza virus infections (1). Unlike TIV, the ability of LAIV to infect cells, replicate, and amplify its antigens enables not only the induction of stronger antibody and CD4^+^ T cell responses, but also antiviral CD8^+^ T cell responses (14–16). Also, since LAIV is delivered intranasally by the same mucosal route used by natural influenza virus infection, the vaccine induces the production of secretory IgA (4, 5). These features allow LAIV to provide 83% efficacy. While LAIV is attenuated, it is contraindicated for use in individuals less than 2 or more than 49 years of age and in pregnant women (17). In 2016, the Centers for Disease Control and Prevention Advisory Committee on Immunization Practices voted that LAIV should not be used during the 2016-2017 flu season, citing lower effectiveness since 2013.

An alternate approach to influenza vaccines is to express the viral genes in *trans* as gene-based vaccines (18, 19). Early efforts at influenza virus gene-based vaccines utilized simple plasmids encoding single influenza virus HA or nucleoprotein genes that were delivered by intramuscular injection or by gene gun in mouse and chicken models (18–20). While the naked DNA vaccines were effective in smaller animals, efficacy waned as these gene-based vaccines were translated into larger animals and humans. Given these efficacy issues, alternate vectors for gene-based vaccination against influenza virus have been tested.

One robust set of gene-based vaccines is adenovirus (Ad) vectors (21–26). Ads are DNA viruses that are also mucosal pathogens. Ad gene-based vaccines can infect the same mucosal surfaces as influenza virus. Therefore, Ads may have utility in educating the mucosal immune system needed to repel seasonal or pandemic influenza viruses (reviewed in reference 27). Compared to other viral vectors, Ad vectors have been shown to drive some of the strongest transgene-specific antibody and CD4^+^ and CD8^+^ T cell responses (22).

While Ad vectors have shown promise as gene-based influenza vaccines, the vast majority of these studies have used replication-defective Ad (RD-Ad) vectors (28–34). When one RD-Ad vector infects a cell, it carries only one copy of an influenza virus antigen and expresses only “1×” protein. RD-Ad antigen expression is directly proportional to the number of infectious virions used. To increase immune responses, one must deliver more vaccine, which also increases the likelihood of side effects. While RD-Ad vaccines have elicited robust protection in small-animal models, like that of plasmid vaccines, Ad efficacy has also waned when scaled up into human trials (35, 36).

An alternate approach is to use replication-competent adenoviral (RC-Ad) vectors to increase influenza vaccine potency (30, 31, 37, 38). Unlike RD-Ad vaccines, each genome of an RC-Ad can be replicated thousands of times in the infected cell, thereby amplifying antigen production per unit virion. This theoretically allows less vaccine to be used to generate the same immune responses as an RD-Ad. However, fully replication-competent Ad vaccines also pose a real safety risk to patients and health care workers, since they can cause adenovirus infections. Therefore, an RC-Ad influenza vaccine may be more potent than RD-Ad but may cause a viral infection in the effort to prevent an influenza virus infection.

We recently described the development of a “single-cycle” Ad (SC-Ad) vector (39, 40). SC-Ads retain their E1 gene to allow them to replicate their DNA, but the expression of key late gene proteins is deleted. In the current best SC-Ad format, the virus “cement” protein pIIIa is deleted in the vector (39, 40). Like RC-Ad vectors, SC-Ads replicate their genomes and any transgenes they carry but do not produce infectious progeny adenovirus virions. In the absence of IIIa, mature virions are not formed and no viral DNA is packaged. We showed that SC-Ad elicits higher and more persistent transgene-specific antibody responses than traditional RD-Ad and RC-Ad vectors in Syrian hamsters and better responses than RD-Ad in rhesus macaques (40). Notably, after single intranasal needle-free administration, mucosal antibody levels climbed over weeks and persisted for more than 6 months in vaginal washes after single intranasal immunization in hamsters (40).

Given that replicating SC-Ad vectors appear to be potent as mucosal vaccines without risk of infection, we tested their use here as needle-free intranasal vaccines against influenza virus. To do this, RD-Ad and SC-Ad vectors based on lower-seroprevalence human adenovirus serotype 6 (Ad6) were modified to express the HA gene from the benchmark influenza A/PR/8/34 virus. These vectors were used to intranasally immunize Ad6-permissive Syrian hamsters. HA binding antibodies were measured as hemagglutination inhibition (HAI) titers as correlates of protection from influenza.

## RESULTS

### RD-Ad6 and SC-Ad6 expressing the hemagglutin cDNA from influenza A/PR/8/34 virus.

**FIG 1:**
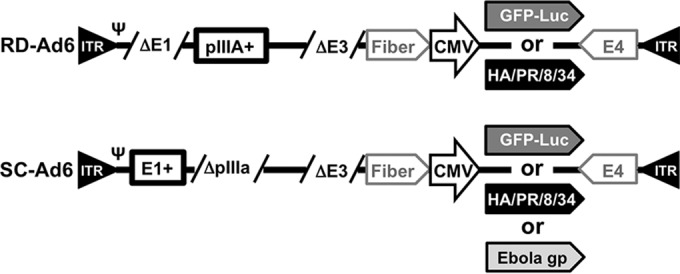
Schematic of Ad genomes expressing influenza A/PR/8/34 virus hemagglutinin. ITR, inverted terminal repeat; Ψ, packaging symbol.

A codon-optimized cDNA for the HA from influenza A/PR/8/34 virus cDNA (34) was cloned into a cytomegalovirus (CMV) expression cassette inserted between Ad6 fiber and E4 genes (Fig. 1). This was recombined in bacteria into the pAd6-ΔE3 plasmid (39, 40). The plasmid was then used to generate RD-Ad6-PR by knocking out the E1 gene and to make SC-Ad6-PR by knocking out the pIIIA gene by recombination. The resulting viral plasmids were confirmed by restriction digest and sequencing. The viral genome insert was liberated from the plasmid and transfected into 293 or 293-IIIA cells for rescue and large-scale virus production.

### RD- and SC-Ad6-PR protein activity in human and hamster cells.

We previously demonstrated that SC-Ad6 vectors expressing a green fluorescent protein (GFP)-luciferase (GL) fusion protein (Fig. 1) generate 100-fold-higher GFP and luciferase activities in human and hamster cells *in vitro* than RD-Ad6 (39, 40). We also demonstrated that SC-Ad-GL generated higher and more persistent antibody responses against GFP in serum and vaginal washes in Syrian hamsters after single intranasal immunization and in rhesus macaques after single sublingual immunization (40).

**FIG 2:**
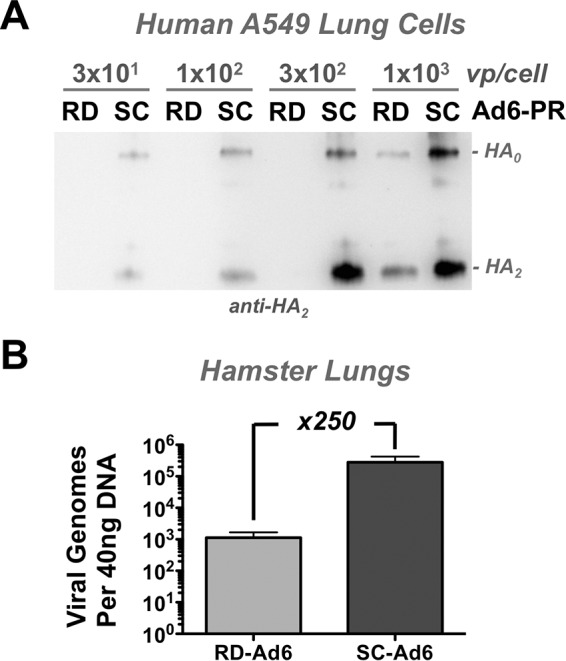
SC-Ad6 antigen expression *in vitro* in human cells and DNA replication *in vivo* in hamster lung. (A) Human A549 lung cells were infected with the indicated virus particle per cell ratios of RD- and SC-Ad6-HA, and cells were harvested 24 h later for Western blotting against the A/PR/8/34 HA antigen. (B) Syrian hamsters were infected intranasally with 1 × 10^11^ vp. On day 3, lung samples were harvested and total DNA was isolated. Viral genome copies were quantified by qPCR. The error bars indicate standard error of the mean (SEM). x250, 250-fold.

To test the ability of RD- and SC-Ad-PR vectors to produce HA antigen, human A549 cells were infected at various multiplicities of infection (MOI) from 30 to 1,000 virus particles (vp)/cell. Twenty-four hours later, the cells were harvested and HA protein was detected by Western blotting with a monoclonal antibody against the A/PR/8/34 HA2 domain (Fig. 2A). Under these conditions, HA protein was detectable after infection with as little as 30 vp/cell of SC-Ad. In contrast, HA was not detectable from the RD-Ad6 vector until 1,000 vp/cell was used in the infection. This 33-fold difference in HA protein production is consistent with the ability to use 33 times fewer virus particles of SC-Ad than RD-Ad to produce equal GFP transduction in primary human airway cells (39, 40). This is also approximately consistent with the ability of SC-Ad to produce 100-fold-higher luciferase levels than RD-Ad *in vitro* in human and hamster cell lines (39, 40).

### RD- and SC-Ad6-PR DNA replication *in vivo* in hamster lungs after intranasal immunization.

One critical aspect of analyzing the effects of replicating Ad vectors is to use host animals that are permissive for the virus. Mice, unfortunately, only weakly replicate human Ad DNA genomes. In contrast, Syrian hamsters support the full adenoviral life cycle and so can actually reflect the effects of genome replication on transgene expression and vaccine responses (40–42).

To test RD- and SC-Ad-PR DNA replication capacity *in vivo*, 10^11^ vp of RD- and SC-Ad6-GL were administered intranasally in groups of 5 Syrian hamsters, and their lungs were harvested 3 days later at the peak of luciferase expression in hamsters (40) for quantitative PCR for viral genomes (Fig. 2B). Under these conditions, animals that received SC-Ad6-GL had 250 times more Ad6 genomes than animals that received the replication-incompetent vector. This indicated that Syrian hamsters can support SC-Ad6 genome and transgene replication needed to test them as influenza vaccines.

### Intranasal vaccination with high-dose SC-Ad6 induces higher levels of serum hemagglutinin antibodies than RD-Ad6 in Syrian hamsters.

**FIG 3:**
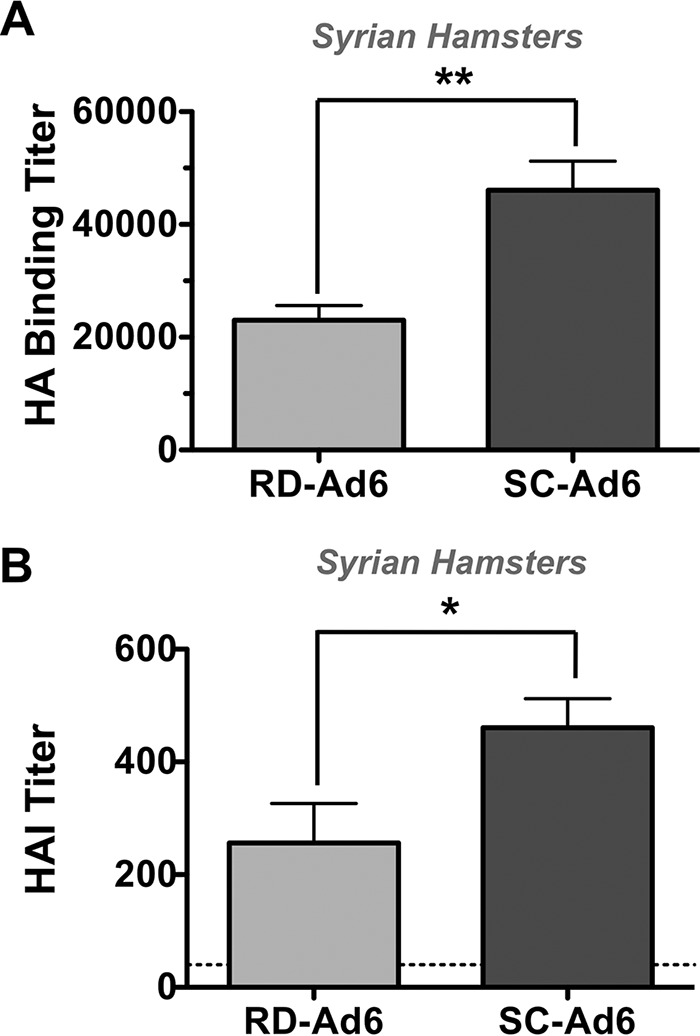
Serum antibody responses following high-dose immunization. Female Syrian hamsters were immunized intranasally with 1 × 10^11^ vp RD- or SC-Ad6-PR. Sera were collected at 7 weeks. (A) Binding antibody titers were measured by ELISA. (B) HAI titers were measured by hemagglutination inhibition assay. The human HAI titer benchmark of 40 is indicated by the dashed line. **, *P* < 0.01; *, *P* < 0.05. The error bars indicate SEM.

Groups of 5 female Syrian hamsters were immunized intranasally with a single dose of 1 × 10^11^ vp of RD- or SC-Ad-HA, and their sera were collected 7 weeks later for antibody assays. HA binding antibody titers were assayed against recombinant influenza A/PR/8/34 virus HA protein by enzyme-linked immunosorbent assay (ELISA) (Fig. 3A). After a single mucosal immunization, SC-Ad6 induced HA binding reciprocal titers in excess of 40,000, significantly higher than those generated by RD-Ad6 (*P* < 0.01).

For influenza vaccine studies, one valuable correlate of protection is the HAI titer. An HAI titer of ≥40 is associated with 50% clinical protection from influenza in young healthy adult humans (43). When the 7-week hamster sera were tested for HAI against A/PR/8/34, SC-Ad6 induced HAI titers more than 10 times higher than the human HAI titer benchmark of 40 (Fig. 3B). These titers were again significantly higher than those generated by RD-Ad6 (*P* < 0.05).

### Single intranasal dose titration of RD- and SC-Ad in hamsters.

**FIG 4:**
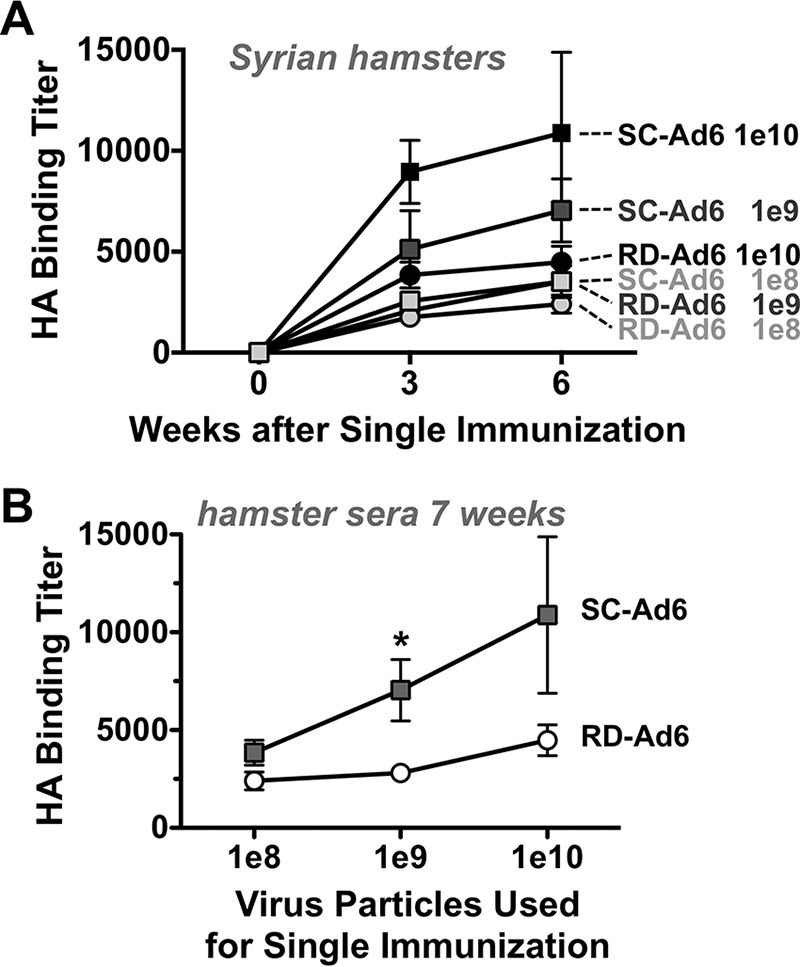
Effect of vaccine dose on the kinetics and levels of serum HA binding antibody titers. Syrian hamsters were immunized intranasally with 1 × 10^8^, 10^9^, or 10^10^ vp of either RD- or SC-Ad6-PR. Samples were collected at weeks 3 and 6. (A) Kinetics of increased HA binding antibody titers over time after single intranasal immunization with the indicated amounts of virus. (B) HA binding titers with dose at week 6. *, *P* < 0.05 by multiple *t* test grouped analysis in Prism. The error bars indicate SEM.

The first experiments utilized a high dose of 1 × 10^11^ vp of Ad vector. This high dose of vaccine might mask differences in the antibody responses generated by the two vectors. To test this, groups of 5 female Syrian hamsters were immunized intranasally a single time with 10^8^, 10^9^, or 10^10^ vp of RD- or SC-Ad6, and HA binding titers were measured 3 or 6 weeks later (Fig. 4A). After single immunization, SC-Ad6 induced consistently higher HA binding titers than RD-Ad6 at every dose. HA binding titers induced by RD- and SC-Ad6 increased with time after single intranasal immunization. However, the titers increased significantly with increasing doses of SC-Ad, while those from the RD-Ad6- immunized animals remained relatively flat despite 100-fold increases in the dose of the vaccine (Fig. 4B).

**FIG 5:**
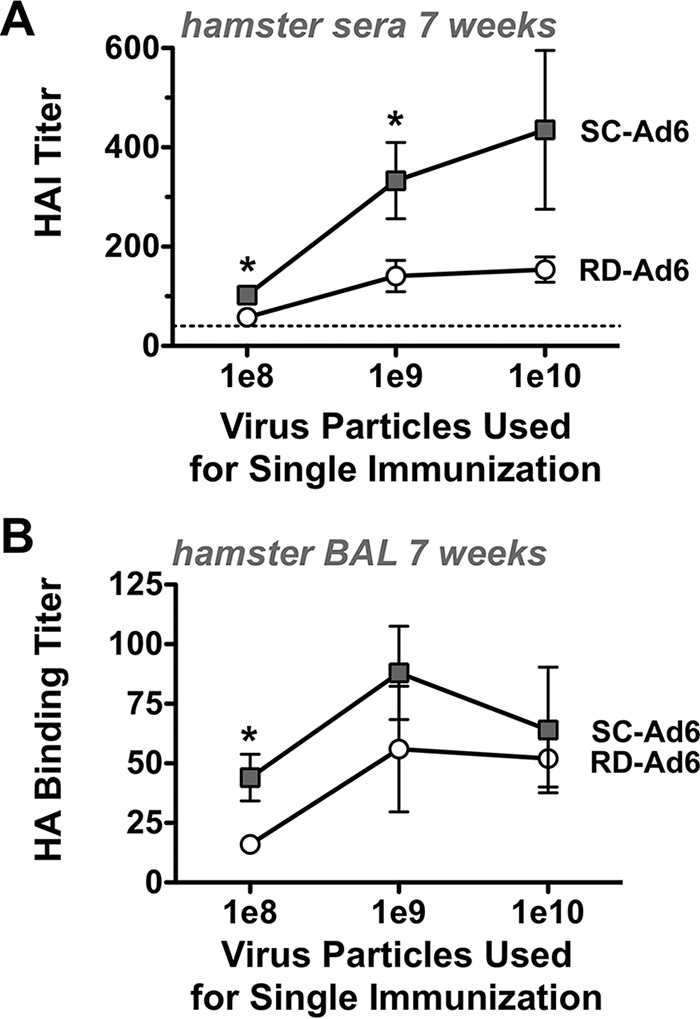
Effect of vaccine dose on week 7 HAI titers in sera and HA binding antibodies in mucosal lung wash samples. Sera and BAL samples were collected at week 7, and binding or HAI titers were measured. (A) A/PR/8/34 HAI titers in serum samples with dose. *, *P* < 0.05 by grouped analysis by multiple *t* test. (B) HA binding titers in BAL samples with dose. *, *P* < 0.05 by grouped analysis by multiple *t* test. The error bars indicate SEM.

At 7 weeks after single immunization, the animals were sacrificed and HA binding and HAI titers were measured in sera and in bronchoalveolar lavage (BAL) fluid (Fig. 5). Notably, the maximal HAI titers generated by RD-Ad at the highest dose of 10^10^ vp were equal to the responses generated by SC-Ad using a 100-fold-lower dose (Fig. 5A). In BAL samples, SC-Ad6 induced higher levels of HA binding antibodies than RD-Ad6 at all doses (Fig. 5B). However, these antibodies were highest at lower vaccine doses. Antibody levels appeared to be attenuated in the BAL fluid at the very highest doses. Titers in the BAL fluid were insufficient for HAI titer testing.

### Single intranasal vaccination with low-dose SC-Ad6 induces higher levels of serum hemagglutinin antibodies than RD-Ad6 in cotton rats.

**FIG 6:**
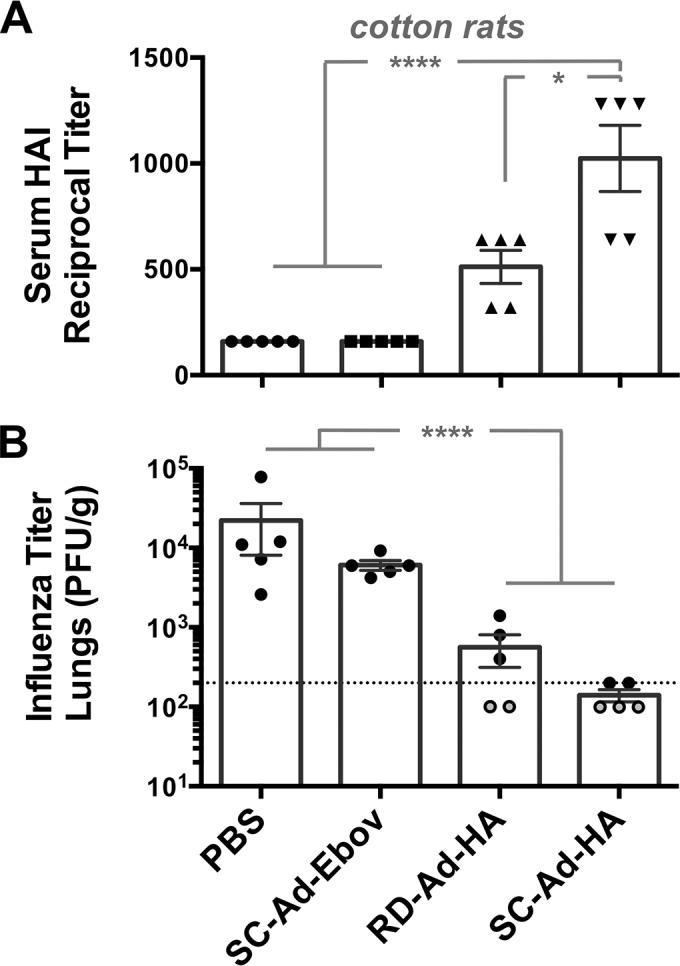
HAI titers and protection in cotton rats after single immunization. Cotton rats were immunized a single time with PBS or 10^9^ vp of RD-Ad6-HA, SC-Ad6-HA, or negative-control vector SC-Ad6-Ebov gp. (A) Sera were collected at week 3, and A/PR/8/34 HAI titers were measured and compared by one-way ANOVA. *, *P* < 0.05; ****, *P* < 0.0001. (B) The animals were challenged with influenza A/PR/8/34 virus, and influenza virus titers were measured in lung homogenates 24 h after challenge. The dotted line indicates the lowest titer of 200 that was tested. The open circles represent samples that had influenza virus titers below this minimal tested dilution of 200 that were given an imputed value of 100. ANOVA was performed on log-transformed values, with pairwise differences (Tukey corrected versions) shown. ****, *P* < 0.0001.

Cotton rats are permissive for both human adenoviruses and human influenza viruses (44, 45). Given this, groups of 5 female cotton rats were immunized intranasally with a single intermediate dose of 1 × 10^9^ vp of RD- or SC-Ad-HA. Phosphate-buffered saline (PBS)-treated animals served as a vehicle negative control. As an Ad vector control, 10^9^ vp of SC-Ad-Ebov gp, expressing an Ebola virus glycoprotein, was administered. Three weeks after single immunization, sera were collected and assayed for influenza A/PR/8/34 virus HAI activity (Fig. 6A). This demonstrated that the two negative-control groups had no HAI activity against influenza A/PR/8/34 virus. RD-Ad6 animals had mean HAI titers of 500, significantly higher than controls (*P* < 0.01 by one-way analysis of variance [ANOVA]). In contrast, single immunization with SC-Ad6 generated HAI titers averaging 1,000, significantly higher than those of the controls (*P* < 0.0001) and significantly higher than those of RD-Ad6 (*P* < 0.05).

### Protection against intranasal influenza virus challenge in cotton rats.

Forty-eight days after single immunization, cotton rats were challenged intranasally with 10^6^ 50% tissue culture infective doses (TCID_50_) of influenza A/PR/8/34 virus. The animals were sacrificed 1 day later, and their lungs were homogenized for influenza virus titration. Control animals vaccinated with PBS had viral loads of 10^3^ to 10^5^ PFU of influenza virus per gram of lung tissue (Fig. 6B). SC-Ad-Ebov gp vector control animals had influenza virus titers of 6 × 10^3^ PFU/g. Influenza virus titers were reduced significantly in both RD- and SC-Ad-vaccinated animals compared to controls (*P* < 0.0001). Mean viral titers in RD- and SC-Ad-PR-immunized animals were 560 and 140 PFU/g, respectively. The viral titers in SC-Ad-vaccinated mice were 200 PFU/g or lower. In contrast, three of five RD-Ad-PR-vaccinated animals had titers above 200 PFU/g. While there were lower titers in individual SC-Ad-vaccinated animals than in all RD-Ad-vaccinated animals, their mean titers did not reach standard significance (*P* = 0.11). These data indicate that gene-based Ad vaccines protect against stringent influenza virus challenge in cotton rats, with significant reductions in viral titers as early as 24 h after high-dose challenge.

## DISCUSSION

Human adenovirus vectors have shown promise as gene-based vaccine platforms for influenza vaccines (23, 29–31, 33, 34, 46). To date, the vast majority of these studies have used RD-Ad vectors. In addition, they have almost all used vectors based on high-seroprevalence Ad5. In this work, we tested if SC-Ad influenza vaccines might be more potent than the standard vectors. In addition, we tested them in the context of lower-seroprevalence human Ad6 vectors (40).

We and others have shown that using a vector that can replicate its genome and the transgene it carries leads to stronger immune responses (47, 48), with these differences especially notable in large animals, including nonhuman primates. Based on this potency, we recently developed SC-Ad vectors. SC-Ad vectors retain their E1 genes and the ability to replicate their viral genomes but have a key structural protein deleted, which prevents the formation of infectious progeny. The first proof of principle for SC-Ad vectors was demonstrated using Ad6 vectors expressing a GFP-luciferase fusion protein (39, 40). SC-Ad6 produced significantly higher reporter gene levels (40) and transgene-specific immune responses than RD-Ad6 in both Syrian hamsters and rhesus macaques (39, 40). Interestingly, SC-Ad vectors were also more potent at generating antibody responses than RC-Ad. In this case, intranasal immunization produced antibody responses that climbed at distant mucosal sites and remained elevated beyond 6 months after single immunization.

Based on these promising results with reporter genes, in this study, we further explored the potential of single-cycle adenoviruses as vaccines against infectious agents using the hemagglutinin gene from influenza A/PR/8/34 virus in Syrian hamsters and cotton rats.

In Syrian hamsters at all doses, SC-Ad6 generated significantly higher levels of antibodies against HA than RD-Ad6. Most telling were the HAI titers driven by SC-Ad6. At all doses, SC-Ad generated HAI titers that were well above the benchmark titer of 40 that is defined as being 50% protective in humans. SC-Ad was able to achieve this titer at doses that were 100-fold lower than the doses needed for RD-Ad6. This 100-fold *in vivo* dose comparison in hamsters corresponds to an amount of RD-Ad 33-fold larger than the amount of SC-Ad that was needed to express equal amounts of GFP in primary human airway epithelial cells (39, 40). Generating antibodies in the lungs has been shown to be particularly beneficial in protecting against influenza, and SC-Ad6 also induces higher levels of antibodies than RD-Ad6 in BAL fluid.

At such a high dose (1 × 10^11^ vp), there is a possibility that the immune responses are due more to a large delivery of vector rather than the effect of genome replication. To test this, Syrian hamsters were vaccinated with a single intranasal dose of 1 × 10^8^, 10^9^, or 10^10^ vp of either RD- or SC-Ad6. SC-Ad6 induced higher binding antibody titers and HAI titers than RD-Ad6 at every dose tested. However, perhaps more notable, SC-Ad6 induces significantly higher HAI titers with each dose increase, while there is no notable difference from the 1 × 10^8^- to 1 × 10^10^-vp doses of RD-Ad6. This suggests the increase in antibodies is due to genome replication and not simply to the delivery of more virus particles.

One key design feature of these studies was the use of Syrian hamsters as the host model for the vaccine. While mice are inexpensive models for influenza, they unfortunately are not permissive for human adenovirus infections (41, 42). In contrast, Syrian hamsters do support Ad6 DNA replication (40), which is key to analyzing the functionality of the single-cycle Ad vaccines. While the hamsters allow SC-Ad to be tested, they likely lead us to underestimate its potency in humans, considering that human Ad6 replicates at least five times less efficiently in hamster HaK cells than in human cells (references 39 and 40 and data not shown). We show here that Ad6 replicates 250-fold in the lungs of hamsters *in vivo* (Fig. 2). This is significantly lower than the capacity with which human Ads can replicate in humans (42, 49), so these results in hamsters likely underrepresent the potential of the single-cycle vaccine platform for human use.

Cotton rats are permissive for both human adenovirus and influenza virus (44, 45). Given this, we tested an intermediate dose of 10^9^ vp of the vaccines delivered a single time by the intranasal route. Within 3 weeks, SC-Ad generated significantly higher HAI titers against influenza virus RD-Ad. These animals were challenged 48 days after single immunization with a high dose of influenza A/PR/8/34 virus, and influenza viral loads in the lungs were measured just 1 day later. Under these stringent challenge conditions, both the RD- and SC-Ad vaccines reduced influenza virus loads in the lungs. Three of the five RD-Ad6-immunized animals had viral titers above 200. In contrast, SC-Ad drove viral levels to 200 PFU/g or less in all the animals. We anticipate that testing in a larger number of animals or with lower doses of vaccine would increase the differences between the two vaccines.

These data in two animal models support the premise that replicating SC-Ad vaccines can mediate stronger immune responses that are beneficial to vaccine protection. Human SC-Ad6 vectors replicate their genomes only 650-fold in hamster cells (40). In contrast, SC-Ad6 replicates its DNA 3,300-fold in human cells (39). This suggests that the increased transgene expression and immune responses that we observed for SC-Ad versus RD-Ad in hamsters and in cotton rats likely underrepresents the potential for increased potency for SC-Ad in humans, where adenovirus genome replication is optimal. This ability of SC-Ad to drive stronger immune responses and to express 33- to 100-fold more transgene protein per unit vaccine (39, 40) may also allow less vaccine to be used in each patient to achieve the same immune responses as RD-Ad. If so, this may have the practical advantage of allowing each good manufacturing practice (GMP) production of SC-Ad to generate more vaccine doses than a parallel production of RD-Ad. These features suggest that single-cycle adenovirus vaccines may hold promise as vaccines against a number of pathogens, including influenza viruses.

## MATERIALS AND METHODS

### Cell culture.

293 human embryonic kidney cells were purchased from Microbix (Toronto, Ontario, Canada). A549 lung carcinoma and Syrian hamster HaK cells were purchased from the American Type Culture Collection (ATCC) (Manassas, VA). All cell lines were maintained in Dulbecco's modified Eagle medium supplemented with 10% fetal bovine serum (FBS) (HyClone, Rockford, IL) and penicillin-streptomycin at 100 U/ml (Gibco). Primary human small airway epithelial cells were purchased from Lifeline Cell Technology (Frederick, MD). They were maintained in BronchiaLife SAE complete medium, also from Lifeline Cell Technology.

### Adenoviruses.

RD-Ad6-PR, SC-Ad6-PR, and SC-Ad6-Ebov gp plasmids were generated in pAd6 plasmid by red recombination in bacteria, as described previously (30, 39, 40). Each virus had its E3 gene cassette deleted and either a codon-optimized influenza virus HA cDNA from A/PR/8/34 or a codon-optimized Ebola virus glycoprotein cDNA cloned into an expression cassette inserted between Ad6 fiber and E4 genes (Fig. 1). The expression cassette consisted of a CMV enhancer driving transcription of the cDNA with a simian virus 40 (SV40) polyadenylation sequence. Viruses were rescued in 293 or 293-IIIA cells as described previously (39, 40) and purified by double CsCl banding. Each virus was desalted in 10% sucrose–potassium phosphate-buffered saline (KPBS). The virus particle concentration was determined by the optical density at 260 nm (OD_260_), and viral infectious units (IFU) were determined with an Adenovirus Rapid Titer kit (Invitrogen) on 293-IIIA cells. The virus particle/IFU ratios for RD-Ad6-PR and SC-Ad6-PR were 228 and 174, respectively.

### In vitro human cell infection and Western blot analysis.

Human A549 lung cells were plated in 6-well dishes and infected the next day at the indicated number of virus particles per cell with the indicated viruses; 24 h later, the medium was removed, and the cells were washed with PBS, harvested, and loaded onto 7.5 to 15% gradient SDS-PAGE Ready Gels (Bio-Rad). Proteins were transferred to nitrocellulose membranes and blocked overnight in 5% milk in Tris-buffered saline with 0.1% Tween 20 (TBST). The blots were incubated with a 1/10,000 dilution of anti-A/PR/8/34 HA2 monoclonal antibody (BEI Resources) and washed in TBST, and then primary antibody was detected with a 1/500 dilution of protein A/G-horseradish peroxidase (HRP) (Pierce). The blots were washed, and HRP was detected using Super Signal West Dura Chemiluminescence reagent on a Kodak In Vivo F instrument.

### Animals.

Female Syrian hamsters (*Mesocricetus auratus*) were purchased from Harlan Sprague-Dawley (Indianapolis, IN). They were housed in the Mayo Clinic Animal Facility under the Association for Assessment and Accreditation of Laboratory Animal Care (AALAC) guidelines, with animal use protocols approved by the Mayo Clinic Animal Use and Care Committee. All animal experiments were carried out according to the provisions of the Animal Welfare Act, the PHS Animal Welfare Policy, the principles of the NIH Guide for the Care and Use of Laboratory Animals, and the policies and procedures of Mayo Clinic. Female cotton rats (Sigmodon hispidus) were bred, housed, and treated at Sigmovir Biosystems, Inc. (Rockville, MD) on a fee for service basis under their IACUC.

### Adenovirus administration to animals.

All viruses were diluted in PBS prior to injection. The indicated dose was diluted to a total of 100 μl, and 50 μl of the solution was administered intranasally into each nare.

### Collection of samples.

Three days after immunization, hamsters were sacrificed and lungs were harvested. Total DNA was isolated using the Maxwell 16 tissue DNA isolation kit (Promega, Madison, WI). Seven weeks postimmunization, hamsters were anesthetized with isoflurane, and blood was collected from the submandibular vein using a 25-gauge syringe in BD microtainer tubes with serum separator (Becton Dickinson and Company). Samples were incubated for 1 h at room temperature, centrifuged at 13,000 × *g* for 2 min, and then transferred to microcentrifuge tubes. Bronchoalveolar lavage samples were collected by exposing the trachea and then injecting 1 ml cold 1% bovine serum albumin (BSA)-PBS into the lungs with a syringe. The liquid was suctioned from the lungs and immediately spun at 13,000 × *g* for 2 min. The supernatant was transferred to a new microcentrifuge tube. All the samples were stored at −20°C until assayed.

### Quantitative real-time PCR (qPCR) of virus DNA in lung tissue.

The concentrations of DNA samples were determined by the OD_260_ and diluted to 20 ng/μl. Real-time PCR was performed using the Applied Biosystems Prism 7900HT sequence detection system with SDS 2.3 software. Each well contained 10 μl Sybr green (Applied Biosystems, Warrington, United Kingdom), 3.8 μl H_2_O, 0.6 μl of 10 μM F primer, 0.6 μl of 10 μM R primer, and 5 μl sample (i.e., 20 ng DNA/well).

### ELISA.

Immulon 4 HBX plates (Thermo, Milford, MA) were coated with 100 ng/well influenza A/PR/8/32 virus HA in 1× PBS (BEI Resources, Manassas, VA) at 4°C overnight. The wells were blocked with 200 μl blocking buffer (0.25% BSA, 0.05% Tween 20, 1× PBS) at room temperature for 1 h. The wells were washed once with sterile water, and 2-fold dilution titers of each sample were prepared. The samples were added to plates and incubated at room temperature for 3 h. The wells were washed 4 times with sterile water. Rabbit anti-hamster IgG-, IgM-, or IgA-HRP (Brookwood Biomedical, Birmingham, AL) was diluted 1:10,000, and 100 μl was added to each well and incubated at room temperature for 2 h. The wells were washed 4 times with sterile water, and 100 μl 1 step Ultra TMB ELISA mixture (Thermo Fisher Scientific Inc., Rockford, IL) was added to each well. When color developed, 50 μl 2 N H_2_SO_4_ was added to each well, and the *A*_450_ was measured using the Beckman Coulter DTX 880 Multimode Detector system.

### Hemagglutination inhibition assay.

Serum samples were mixed with RDE (II) Seiken (Denka Seiken Co., Tokyo, Japan) at a 1:3 ratio. Samples were incubated at 37°C for 20 h and then inactivated at 56°C for 60 min. Twofold dilutions were prepared in 0.5% BSA-PBS in 96-well round-bottom plates. Influenza A/PR/8/34 virus was diluted to 8 hemagglutinating units (HAU) and added to each sample in the plate except antigen control wells. The plates were incubated for 30 min. Chicken red blood cells (CRBC) were washed 2 times with PBS, diluted to a 1% CRBC suspension, and added to each well in the plate. The plates were incubated for 30 min, and the agglutination patterns were read.

### Influenza A/PR/8/34 virus challenge and titration.

Cotton rats were challenged intranasally with 10^6^ TCID_50_ of influenza A/PR/8/34 virus. One day later, lung homogenates were clarified by centrifugation and diluted 1:10 and 1:100 in Eagle's minimal essential medium (EMEM). Confluent MDCK monolayers in 24-well plates were infected in duplicate with undiluted (neat) samples, followed by diluted homogenates. After 1 h incubation at 37°C in a 5% CO_2_ incubator, the wells were overlaid with 2% agarose in EMEM-3.75% BSA supplemented with 1.0 μg/ml tosylsulfonyl phenylalanyl chloromethyl ketone (TPCK)-trypsin, and the plates were returned to the 37°C incubator. After 7 days of incubation, the overlay was removed, and the cells were fixed with 0.1% crystal violet stain for 1.5 h and then rinsed and air dried. The plaques were counted, and the viral titers were expressed as PFU per gram of tissue.

### Data analysis.

Statistical analyses were performed using Prism Graphical and JMP software.
